# Identifying Communities at Risk for COVID-19–Related Burden Across 500 US Cities and Within New York City: Unsupervised Learning of the Coprevalence of Health Indicators

**DOI:** 10.2196/26604

**Published:** 2021-08-26

**Authors:** Andrew Deonarine, Genevieve Lyons, Chirag Lakhani, Walter De Brouwer

**Affiliations:** 1 XY.ai Cambridge, MA United States

**Keywords:** COVID-19, satellite imagery, built environment, social determinants of health, machine learning, artificial intelligence, community, risk, United States, indicator, comorbidity, environment, population, determinant, mortality, prediction

## Abstract

**Background:**

Although it is well-known that older individuals with certain comorbidities are at the highest risk for complications related to COVID-19 including hospitalization and death, we lack tools to identify communities at the highest risk with fine-grained spatial resolution. Information collected at a county level obscures local risk and complex interactions between clinical comorbidities, the built environment, population factors, and other social determinants of health.

**Objective:**

This study aims to develop a COVID-19 community risk score that summarizes complex disease prevalence together with age and sex, and compares the score to different social determinants of health indicators and built environment measures derived from satellite images using deep learning.

**Methods:**

We developed a robust COVID-19 community risk score (COVID-19 risk score) that summarizes the complex disease co-occurrences (using data for 2019) for individual census tracts with unsupervised learning, selected on the basis of their association with risk for COVID-19 complications such as death. We mapped the COVID-19 risk score to corresponding zip codes in New York City and associated the score with COVID-19–related death. We further modeled the variance of the COVID-19 risk score using satellite imagery and social determinants of health.

**Results:**

Using 2019 chronic disease data, the COVID-19 risk score described 85% of the variation in the co-occurrence of 15 diseases and health behaviors that are risk factors for COVID-19 complications among ~28,000 census tract neighborhoods (median population size of tracts 4091). The COVID-19 risk score was associated with a 40% greater risk for COVID-19–related death across New York City (April and September 2020) for a 1 SD change in the score (risk ratio for 1 SD change in COVID-19 risk score 1.4; *P*<.001) at the zip code level. Satellite imagery coupled with social determinants of health explain nearly 90% of the variance in the COVID-19 risk score in the United States in census tracts (*r*^2^=0.87).

**Conclusions:**

The COVID-19 risk score localizes risk at the census tract level and was able to predict COVID-19–related mortality in New York City. The built environment explained significant variations in the score, suggesting risk models could be enhanced with satellite imagery.

## Introduction

The COVID-19 pandemic has disrupted major world economies and overwhelmed hospital intensive care units worldwide [1]. In the United States alone, the virus has spread throughout urban and rural communities and killed over 300,000 Americans to date [2]. Case series and epidemiological surveillance data from the United States [3-6] and around the world [7-11] have implicated risk factors for COVID-19–related morbidity and mortality, including older age, male sex, impaired lung function, cardiometabolic-related diseases (eg, diabetes, heart disease, or stroke), and obesity. In the United States, comorbidities are known to cluster in geographies such as the southeast states and counties (eg, in chronic disease [12] and in COVID-19 [13-16]), and are partly mediated by built environment features, such as walkability [17]. Although race and ethnicity have been identified as risk factors, systemic racism and discrimination in the health care system play an important role in this relationship [18-20]. Additionally, racial and ethnic discrimination have influenced where individuals reside and has played a substantial role in the increased morbidity and mortality related to COVID-19 [21]. Other factors including the built environment and air pollution have been associated with COVID-19 infection and complications [22,23], but it has been unclear how to prioritize these associations to prevent complications. Both individual-level factors (eg, diabetes, smoking, and asthma [3,8,10,11]) and geographical-level social determinant factors (eg, census tract–level population density and increased household occupancy) are strong risk factors for COVID-19 infection and risk [24]. Social determinants of health are defined as “conditions in the environments where people are born, live, learn, work, play, worship, and age that affect a wide range of health, functioning, and quality-of-life outcomes and risks” [25]. Social determinants of health can be grouped into five domains, including economic stability, education access and quality, health care access and quality, neighborhood and built environment, and social and community context [25]. Recently, Maharana and Nsoesie [26] developed an approach to map the built environment to obesity prevalence using deep learning analysis of satellite imagery, highlighting a potentially novel method of using measurements of the built environment to quantify disease risk.

At the time of writing, New York emerged as a location with several COVID-19–related deaths spread across the 2141 census tracts in the city. Even within city hot spots like New York City, common chronic diseases and their risk factors for COVID-19 are geographically heterogeneous and vary per unit of geography, including within and across states, counties, and even cities. It is unclear how the heterogeneity of community-based risk or prevalence of diseases at a census tract level (median population sizes of ~3000-5000 individuals) is related to COVID-19 risk. Furthermore, analyses on coarser spatial resolutions will attenuate predictions and associations [27].

In this investigation, we sought to create a clinically focused risk score that could be used to predict COVID-19 cases and deaths within cities, identify hot spots at the subcounty (census tract) level, and identify potentially vulnerable communities, and to determine how the social determinants of health and the built environment may explain the variance of this clinically focused risk score and whether the built environment explains statistically significant amounts of score variance even after accounting for the social determinants of health. To do this, we developed the COVID-19 community risk score (COVID-19 risk score) that summarizes the complex comorbidity and demographic patterns of small communities at the census tract level. Additionally, we examined how the social determinants of health (including the built environment, measured using satellite imagery methods [26]) explained score variance and validated the risk score by examining its relationship with zip code–level deaths during the late-May 2020 COVID-19 epidemic in New York City. Last, we deployed the COVID-19 risk score with an application programming interface and a browsable dashboard [28].

## Methods

### Study Data

**Figure 1:**
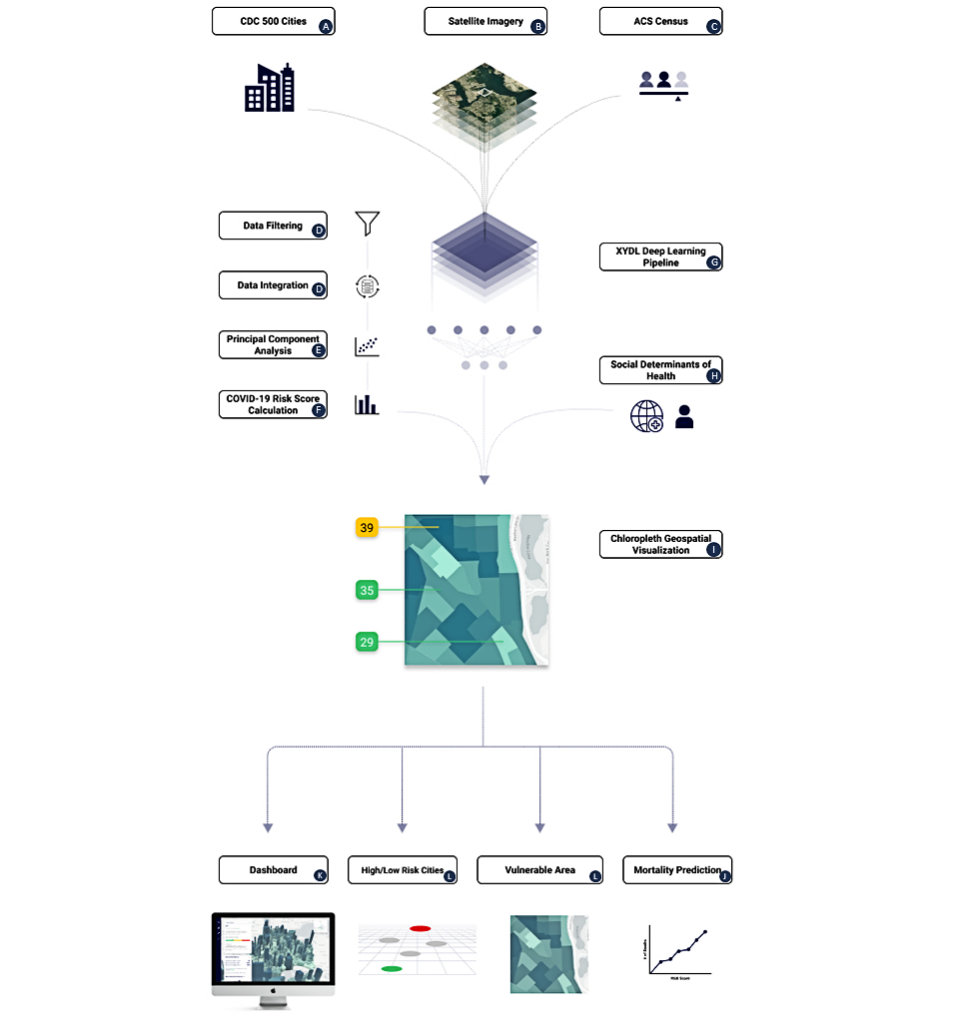
Overview of study. (A) CDC 500 Cities; (B) satellite imagery of 500 cities from OpenMapTiles; (C) ACS Census summary statistics for each census tract; (D) estimates of prevalence and coprevalence of disease and health indicators for risk of COVID-19 complications; (E) use of principal components analysis to reduce dimensionality of diseases and health indicators; (F) construction of COVID-19 score from principal components; (G) “XYDL” deep learning pipeline that inputs satellite imagery, social determinants of health indicators from ACS Census data to predict COVID-19 community risk score; (H) social determinants of health from ACS Census data; (I) visualization of the COVID-19 community risk score; (J) association of the COVID-19 risk score with mortality in New York City; (K) creation of a dashboard; (L) mapping highest and lowest risk cities and tracts as a function of the risk score. ACS: American Community Survey; CDC: Centers for Disease Control and Prevention.

We obtained geocoded disease prevalence data at the census tract level from the US Centers for Disease Control and Prevention (CDC) 500 Cities Project (the December 2019 release, which is based on data from 2016 to 2017 [29]; Figure 1A). The project 500 Cities contains disease and health indicator prevalence for 27,648 individual census tracts of the 500 largest cities in the United States, and these prevalences are estimated from the Behavioral Risk Factor Surveillance System [30].

From the 500 Cities data, we chose 13 population-level health indicators that correspond to individual-level chronic disease risk factors associated with COVID-19–related hospitalization and death based on reports from China, Italy, and the United States (eg, [3,8,10,11]). Disease indicators include the prevalence among adults of diabetes, coronary heart disease, chronic kidney disease, asthma, arthritis, any cancer, or chronic obstructive pulmonary disorder. We also selected behavioral risk factors including smoking and obesity, and the prevalence of individuals on blood pressure medication. We chose these comorbidities and risk factors with guidance from the CDC because they were classed as among the strongest risk factors for COVID-19–related hospitalization, intensive care unit use, and death (eg, males and females older than 65 years, diabetes, heart disease, and stroke); were indicative of risk for cardiometabolic disease or impaired lung function, which are risk factors for COVID-19 (eg, smoking, obesity, high blood pressure, high cholesterol, kidney disease, asthma, or chronic pulmonary obstructive disorder); or involve pharmacological interventions that could result in an immunocompromised state (eg, certain antineoplastic, arthritis, and antihypertensive medications) [31].

We further obtained 5-year 2013-2017 American Community Survey (ACS) Census data [32], which contain sociodemographic prevalences and median values for census tracts (Figure 1C), and corresponded to the 2016-2017 CDC 500 Cities data. We also selected the total number of individuals in the tract, proportion of males and females older than 65 years, and proportion of individuals by race and ethnicity, which included African American, Mexican, Hispanic, Asian, and White groups from the ACS. Race and ethnicity were examined to determine if there were different risks associated with these groups (where race is a socially constructed concept that can be used as a proxy for the complex interplay of institutional and individual-level racism and barriers to health care experienced by these different groups [33]). These data also included information on socioeconomic indicators including median income, the proportion of individuals living in poverty, unemployment, cohabitation with more than one individual per room, and having no health insurance. These measures were previously identified as possible contributors to increased risk of infection or mortality associated with COVID-19 [20,34].

### Defining the COVID-19 Community Risk Score

Given the complex interplay between the social determinants of health, chronic disease, and the built environment, we sought to first examine how clinical comorbidities could be used to predict COVID-19 rates by developing a clinically focused risk score and then examine how these comorbidities relate to the built environment and social determinants of health. Understanding if the built environment and social determinants of health can explain the variance of a clinically focused risk score would show that more complex risk models could be built using this data in the future. To do this, we used the statistical programming language R (version 4.0.5; R Foundation for Statistical Computing) [35] to merge disease and behavior prevalence data from the CDC 500 Cities Project for each of the 27,648 census with ACS information and calculate their Pearson pairwise correlations (Figure 1D) to determine how the data were correlated with each other. We considered 15 variables in total, including 13 health indicators (eg, diseases and risk factors), and 2 demographic factors, the proportion of male and female individuals older than 65 years in the risk score. The disease prevalence included any form of cancer, arthritis, stroke, chronic asthma, chronic obstructive pulmonary disease (COPD), heart disease, diabetes, kidney disease, high blood pressure, and high cholesterol. Behavioral and lifestyle-related risk factors included smoking, obesity, and the rate of individuals on blood pressure medication. Finally, demographic factors included the prevalence of males and the prevalence of females older than 65 years.

### Socioeconomic Correlates of the Community COVID-19 Risk Score

Next, we examined the relationship between the ACS-derived sociodemographic indicators with the COVID-19 risk score. This was done by calculating multivariate linear and random forest regressions to test the linear and nonlinear contribution of the sociodemographic indicators in the COVID-19 score (Figure 1H), and provide insight into the relationship of sociodemographic factors and the clinical indicators used in the COVID-19 score. This comparison to sociodemographic factors also serves as a form of validation, as the risk increases, one would expect certain sociodemographic indicators to also increase, such as poverty. Further details concerning the calculation of the linear and random forest regression can be found in Multimedia Appendix 1 [28,35-38].

### Association of the COVID-19 Community Risk Score With Satellite Imagery

To correlate the COVID-19 risk score from satellite imagery (Figure 1B), millions of satellite images (n=4,742,919) were analyzed in an ensemble of an unsupervised deep learning algorithm and a supervised machine learning algorithm. The images are satellite raster tiles that were downloaded from the OpenMapTiles database. The images have a spatial resolution close to 20 meters per pixel, allowing a maximum zoom level of 13 [39]. Images were extracted in tiles from the OpenMapTiles database using the coordinate geometries of the census tracts. After extraction, images were digitally enlarged to achieve a zoom level of 18.

Many census tracts are large enough to contain multiple satellite images. The median number of images per tract was 94, and the number of images per census tract ranged from 1 image in the census tract to the largest geographical tract with 162,811 images (in Anchorage, Arkansas) with an IQR from 43 to 182 images. The geographical coverage of the images per census tract ranged from the smallest census tract covering 0.022 km^2^ and the largest census tract covering 5679.52 km^2^, with an IQR from 0.93 km^2^ to 3.89 km^2^ and a median of 1.92 km^2^ per census tract.

First, using the Python 3.7.7 programming language [40], we passed images through AlexNet [41], a pretrained convolutional neural network, in an unsupervised deep learning approach called feature extraction [42] (Figure 1G). The resulting vector from this process is a *latent space feature* representation of the image comprising 4096 features. This latent space representation is essentially an encoded (non–human readable) version of the visual patterns found in the satellite images, which, when coupled with machine learning approaches, is used to model the built environment of a given census tract [26]. For each census tract, we calculated the mean of the latent space feature representation. We performed feature extraction on a NVIDIA Tesla T4 GPU using the PyTorch package in Python. Finally, the latent space feature representation was regressed against the COVID-19 risk score variance using gradient boosted decision trees [43]. We deployed existing AlexNet deep learning models originally trained on images from the internet and fine-tuned [44] them to predict the variance associated with the COVID-19 risk score, framing the analysis as a regression task. To do this, we split the census tract data set (with the split being fully randomized) into 80:20 and 50:50 training and testing groups to get a conservative estimate of variance explained and predictive capability of the sociodemographic variables in the COVID-19 risk score while not overfitting the data. To train the model, we used a maximum tree depth of 5, a subsample of 80% of the features per tree, a learning rate (ie, feature weight shrinkage for each boosting step) of 0.1, and used threefold cross-validation to determine the optimal number of boosted trees. Training was completed on a NVIDIA Tesla T4 GPU using Python 3.7.7 and the XGBoost package. In a separate analysis, both satellite image features and the social determinants of health features (previously mentioned) were regressed against the COVID-19 risk score variance. We reported R^2^ for the predictions in the test data (Figure 1G, 1H).

### Association of the COVID-19 Community Risk Score With Zip Code–Level COVID-19–Attributed Mortality

We downloaded case and death count data on a zip code tabulation area (ZCTA) of New York City, a hot spot of the US COVID-19 epidemic as of May 20, 2020, and then again on September 20, 2020 (Figure 1J). We used 2010 census crossover files to map census tracts to ZCTAs. We mapped the COVID-19 risk score to each ZCTA in New York City in April and September 2020. Each ZCTA had information on the total number of COVID-19 tests, positive cases, and COVID-19–related deaths. We computed the average COVID-19 risk score for the ZCTA, weighting the average by population size of the census tract. As previously mentioned, we estimated the ZCTA-level socioeconomic values and proportions. We associated the COVID-19 risk score with the death rate using a negative binomial model. We set the offset term as the logarithm of the total population size of a zip code. The exponentiated coefficients are interpreted as the incidence rate ratio for a unit change (eg, 1 SD increase) in the variable (vs no change). We also examined multicollinearity, calculating the variance inflation factor (VIF) using the VIF function in the regclass package in R.

### Data Availability Through the COVID-19 Risk Score Application Programming Interface and Dashboard

Finally, the COVID-19 risk score was made publicly available through an application programming interface and online web dashboard (see Multimedia Appendix 1).

### Ethics Approval

Ethics approval was not required for this investigation as the study did not involve any human participants, and all of the data used were obtained from publicly available data sets.

## Results

### Prevalence and Heterogeneity of COVID-19–Associated Comorbidities and Risk Factors Across 500 Cities of the United States

**Figure 2:**
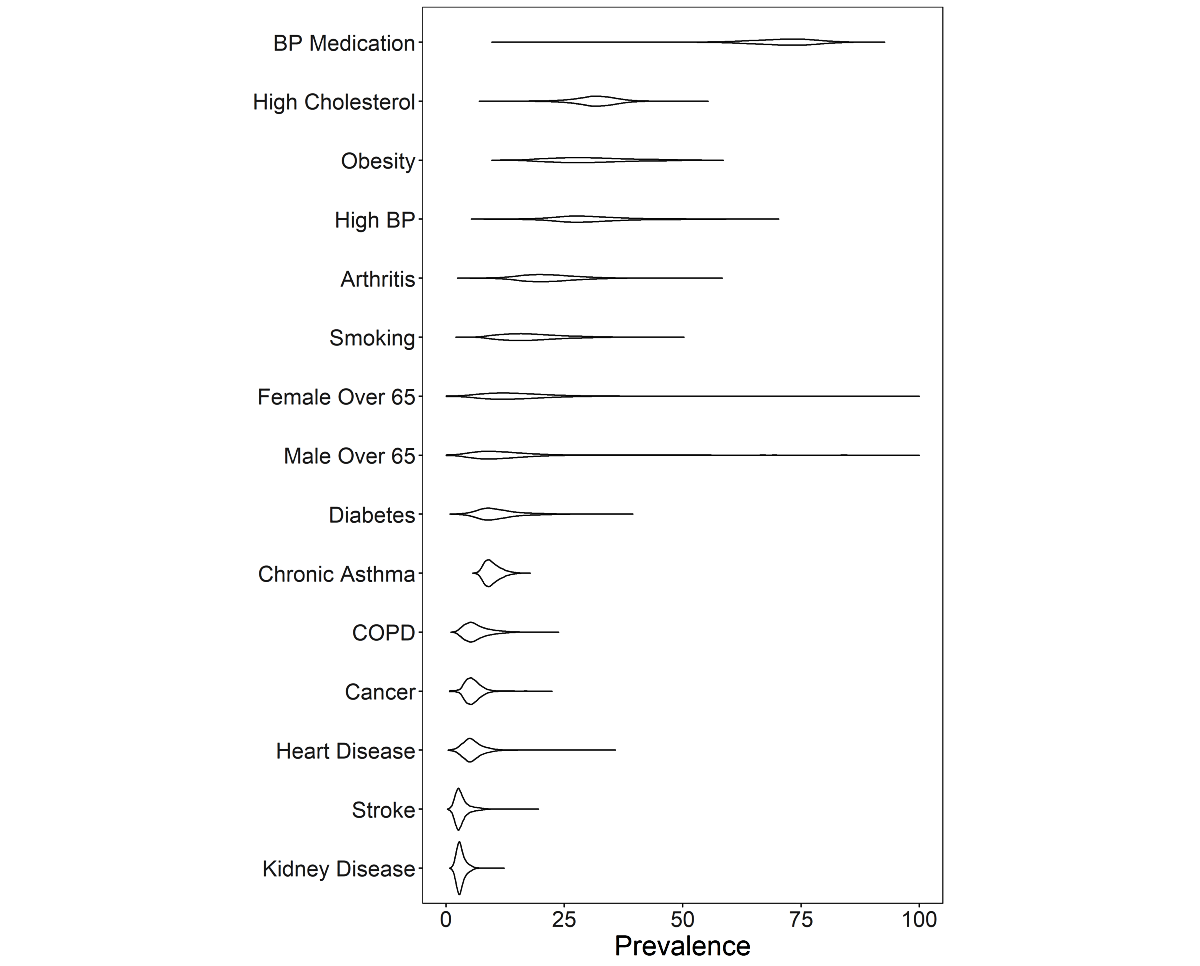
Per census tract prevalence for health indicators (y-axis). BP: blood pressure; COPD: chronic obstructive pulmonary disease.

**Figure 3:**
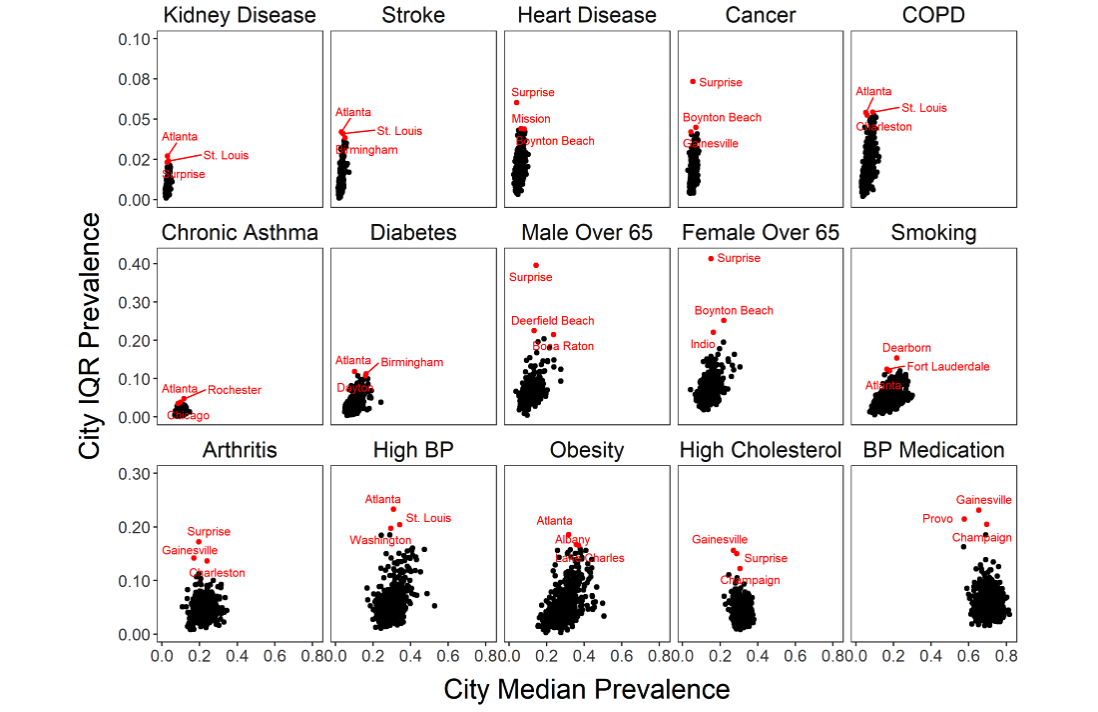
Median prevalence within a city versus the IQR of the prevalence of health indicators (top 3 cities with the largest IQR are highlighted in red). BP: blood pressure; COPD: chronic obstructive pulmonary disease.

We present summary statistics of the prevalence of the 15 COVID-19 comorbidities and risk factors for 27,648 census tracts across the United States using the 2019 release of the CDC 500 Cities data (derived from data obtained in 2017) and ACS data collected between 2013 and 2017 (Figure 1A, 1C). Census tracts represent small *communities* that have a median population size of 4091 (total range of 15-51,536). From the 500 cities analyzed, there was a median number of 28 (IQR 20-47) census tracts, with the most tracts found in New York (2141 tracts, with a population of n=8,440,712), Los Angeles (992 tracts, with a population of n=3,961,681), and Chicago (794 tracts, with a population of n=2,726,431), while Meridian, Idaho (4 tracts, with a population of n=53,442) has the fewest number of tracts. There was a wide range of prevalence values (ranging from 6% to 100%; Figure 1D, Figure 2) for the different prevalence measures, and a wide range of IQR values within cities was noted (Figure 3 and Tables S1 and S2 in Multimedia Appendices 2 and 3).

Atlanta had the greatest IQR for obesity (22%-40%), high blood pressure (20%-44%), and COPD (4%-9%), while Gainesville had the highest variation in prevalence of high cholesterol (18%-34%) and blood pressure medication (51%-74%).

### Comorbidity and Risk Factor Patterns Across the United States

**Figure 4:**
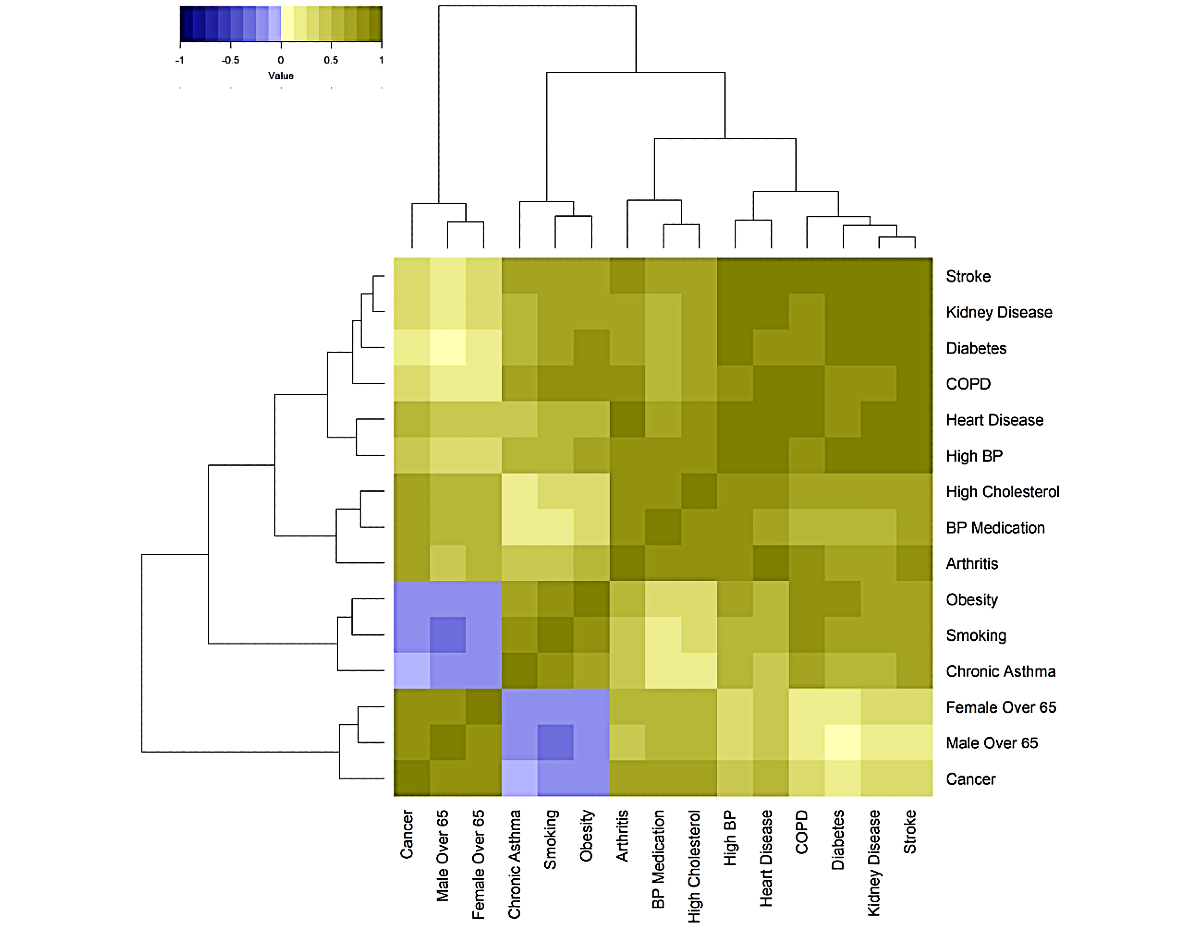
Pearson correlation of health indicators across 27,648 census tracts (legend value corresponds to Pearson correlation value). BP: blood pressure; COPD: chronic obstructive pulmonary disease.

The Pearson correlations between the 15 different prevalence values was calculated using census tract–level data (Figure 1D, Figure 4), with a median absolute value of correlation of 0.63 (IQR 0.35-0.78) noted with disease prevalences. The mean pairwise correlation between cardiometabolic diseases (diabetes, stroke, and heart disease) was 0.92, for cardiovascular risk factors (obesity, high blood pressure, and high cholesterol) was 0.62, and for smoking and respiratory conditions (asthma and COPD) was 0.69. An average correlation of 0.78 existed for diseases like diabetes, stroke, and heart disease, with obesity highly correlated with all of them (mean correlation 0.54), and a mean correlation of 0.78 was found for males and females older than 65 years and cancer prevalence.

**Figure 5:**
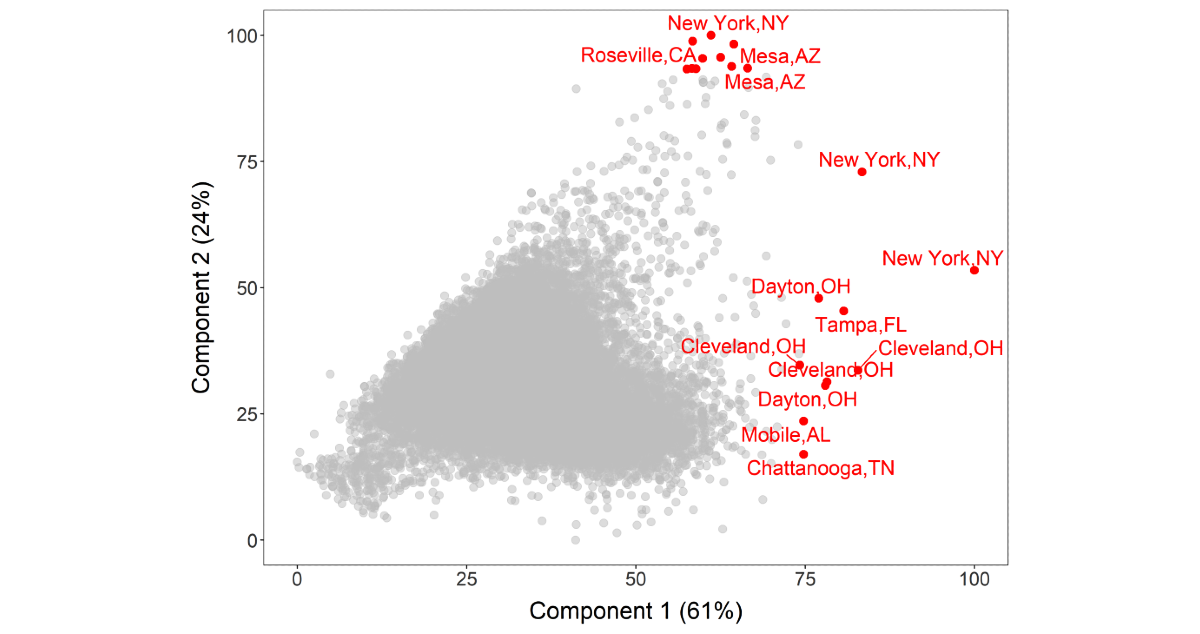
Scatterplot showing the relationship between the first and second principal components from principal component analysis, with each point indicating a city or census tract in the United States (top 10 cities/tracts by principal component 1 or 2 are highlighted in red).

The first two principal components of the 15 COVID-19 health indicators and risk factors described 85% of the total variation (61% and 24% for component 1 and 2, respectively, see Figure 5) of the variation over all 27,648 census tracts (Figure 1E). The first principal component had equal contribution from all 15 health indicators and risk factors, except for cancer and males and females older than 65 years; the second principal component was dominated by cancer and age (Table S3 in Multimedia Appendix 2). This pattern of health indicator and risk factor contribution to principal components was also noted when the COVID-19 risk score was calculated at the city and county level (Table S3 in Multimedia Appendix 2).

### Calculating a Robust COVID-19 Community Risk Score

The COVID-19 risk score was calculated using the 15 disease and health indicators for 27,648 included census tracts. The average score was 33.7 (SD 8.6); the median was 33.32 (IQR 28-38). Table 1 shows the communities with the highest variation of scores in the United States. The average error of the COVID-19 risk score across the census tracts was 1.25 (SD 0.85).

**Table 1:** Cities with the largest variation of COVID-19 risk score.

City, State	Median	Min	Max	25th percentile	75th percentile	SD	IQR
Athens, GA	32.2	6.3	42.6	20.7	35.7	10.0	15.0
Atlanta, GA	33.7	4.7	53.7	23.8	41.4	11.0	17.6
Boynton Beach, FL	41.7	21.6	81.3	35.5	52.1	16.2	16.6
Champaign, IL	29.8	3.4	45.2	17.9	34.1	12.8	16.2
Gainesville, FL	27.1	2.2	75.2	16.6	38.5	15.0	21.9
Hemet, CA	41.2	30.1	67.9	36.4	53.4	11.1	17.0
Mesa, AZ	32.2	7.7	84.6	29.0	43.2	14.7	14.2
Montgomery, AL	41.0	15.0	61.3	33.5	47.6	9.8	14.1
St. Louis, MO	36.9	22.0	57.3	31.7	46.3	8.6	14.6
Surprise, AZ	30.2	24.1	77.9	26.1	58.7	20.6	32.6
Birmingham, AL	43.9	18.8	57.9	36.4	49.3	9.6	12.9
Cape Coral, FL	43.4	30.3	63.3	37.3	49.3	8.2	12.0
Clearwater, FL	42.9	28.7	66.4	39.6	51.5	8.0	12.0
Cleveland, OH	42.4	18.3	78.3	38.0	49.6	9.1	11.6
Dayton, OH	43.1	6.0	78.1	38.6	49.8	11.8	11.2
Huntsville, AL	42.7	22.1	56.3	32.9	45.6	8.4	12.7
Lake Charles, LA	41.5	27.4	54.0	36.4	46.5	7.5	10.1
Lakeland, FL	43.9	18.0	65.8	38.3	49.2	10.9	10.9
Largo, FL	45.0	26.1	75.3	41.1	53.2	11.6	12.2
Palm Coast, FL	46.8	33.9	58.1	43.7	54.8	7.5	11.0
Pompano Beach, FL	43.6	27.1	64.3	37.0	48.5	9.7	11.5
Shreveport, LA	42.8	21.7	64.0	37.7	49.7	8.6	12.1
Gary, IN	50.8	42.5	61.8	47.1	54.6	5.2	7.6

### COVID-19 Community Risk Score Variance Can Be Explained by Social Determinants of Health and Satellite Images of the Built Environment

The social determinants of health measures (excluding built environment) and demographic characteristics of a community (Figure 1C, 1H) explain 54% of the total additive variation calculated using multiple linear regression (*r*^2^=0.54; *P*<.001) of the COVID-19 risk score in the testing data set (when using a 50:50 and 80:20 fully randomized training:testing split). In this regression analysis, low to moderate multicollinearity was found with VIFs ranging from 1.41 for the variable *not employed* to 4.71 for *less than high school*. We found an additional 11% of variation attributed to nonlinear relationships, or a total of 65% between social determinants and the COVID-19 risk score, in the testing data using random forest–based regression (*r*^2^=0.65; *P*<.001). The built environment features captured by satellite images contributed to 27% of the variation in the COVID-19 risk score. In total, combining both social determinants and satellite imagery explained 87% of the variation of the COVID-19 risk score when using an 80:20 training:testing split (Figure 1G, 1H).

Concerning important features, all 13 sociodemographic variables correlated with the COVID-19 risk score (linear regression *P*<.001 for 11 out of 13 variables) illustrated in Table 2. The variables that had the largest additive contribution included the proportion of the community that was nonemployed (for a 1 SD change in proportion of nonemployed was associated with a 5.3 unit increase in the COVID-19 score; *P*<.001). A 1 SD increase in the increase of individuals with less than a high school education was associated with a 2 unit increase in the score. However, a 1 SD change in the increase of those at or below the poverty level was associated with a 3.3 unit decrease in the COVID-19 risk score. We found low to moderate VIFs associated with each sociodemographic variable (Table 2).

When assessing the explained variance using nonlinear regression (random forest) methods, the *most important* variables in the training data (ascertained through a permutation of each variable sequentially) included the proportion of the tract that was not employed (273% increase of mean squared error [MSE] when permuted), of Asian ethnicity (93% increase of MSE), at or below poverty (91% increase of MSE), Hispanic (78% increase MSE), and less than high school (78% increase MSE). The rank order of the importance of these variables was similar to the strength of their association in the linear model (Table 2). The same results were observed when the training:testing split was 50:50 and 80:20.

**Table 2:** Multivariate coefficients and CIs for linear regression and random forest regression of the COVID-19 risk score.

Variable	Linear coefficient	*P* value	Low (95% CI)	High (95% CI)	MSE^a^	Node purity^b^	VIF^c,d^
Median income	–1.34	<.001	–1.53	–1.16	42	59,736	3.68
Median home value	–0.13	.07	–0.27	0.01	39	33,163	2.21
At or below poverty (%)	–3.24	<.001	–3.42	–3.07	61	78,890	3.04
Unemployment (%)	0.73	<.001	0.60	0.86	87	68,364	1.69
Nonemployed (%)	5.38	<.001	5.26	5.50	285	316,903	1.42
Less than high school (%)	2.12	<.001	1.90	2.33	71	63,048	4.71
No health insurance (%)	0.69	<.001	0.55	0.83	50	34,818	2.18
More than 1 occupant (%)	–0.89	<.001	–1.04	–0.73	59	41,387	2.46
African American (%)	0.73	<.001	0.59	0.87	68	84,497	2.09
Hispanic (%)	–2.30	<.001	–2.49	–2.10	78	63,847	4.12
Asian (%)	–1.14	<.001	–1.25	–1.02	91	93,675	1.42
Other race (%)	–0.51	<.001	–0.67	–0.36	69	45,301	2.45

^a^MSE: mean standard error.

^b^Node impurity: residual sum of squares for the random forest model.

^c^VIF: variance inflation factor.

^d^For the linear regression model.

### COVID-19 Community Risk Score Was Associated With COVID-19 Death Rate in New York City

**Figure 6:**
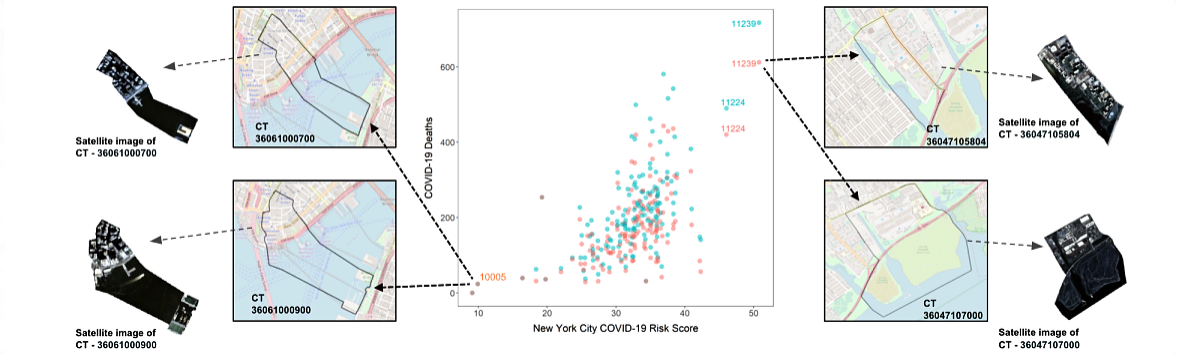
COVID-19 deaths as a function of the COVID-19 risk score in New York City for each zip code (middle panel). The zip codes with the highest and lowest death rates are annotated. Blue points denote data on the epidemic death counts in September 2020. Red points denote epidemic death counts in May 2020.

A 1 SD increase in the COVID-19 risk score was associated with a 40% increase in the incident rate ratio (IRR 1.40 per 1 SD increase; *P*<.001; Figure 6 and Table 3) in both May and September 2020. For zip codes (eg, Figure 6 annotated zip codes) that had COVID-19 risk scores greater than 40, there was an almost twofold increase in death rates (IRR 1.98, 95% CI 1.43-2.77; *P*<.001). Additionally, we assessed multicollinearity by calculating the VIFs for each variable and found moderate to high multicollinearity.

**Table 3:** Multivariate incidence rate ratios (for 1 SD change in the variable) for zip code–level deaths in New York City in May and September 2020.

Variable (per 1 SD unit)	May IRR^a^ (95% CI)	May *P* value	VIF^b^	September IRR (95% CI)	September *P* value	VIF
COVID-19 risk score	1.40 (1.27-1.55)	<.001	2.20	1.40 (1.27-1.53)	<.001	2.20
Median income	1.02 (0.84-1.22)	.80	9.06	0.99 (0.82-1.18)	.90	9.12
Less than high school	0.81 (0.008-1.81)	.10	19.80	0.81 (0.62-1.06)	.10	19.64
College educated	0.93 (0.26-1.92)	.50	10.83	0.93 (0.76-1.14)	.50	10.77
African American	1.14 (1.03-2.78)	.03	3.91	1.16 (1.03-1.30)	.01	3.95
Mexican	0.9 (0.87-1.08)	.60	3.72	0.97 (0.87-1.07)	.50	3.73
Hispanic	1.27 (1.19-1.46)	<.001	5.60	1.29 (1.12-1.47)	<.001	5.60
Asian	1.12 (1.00-1.26)	.05	4.34	1.15 (1.02-1.28)	.02	4.34
At or below poverty	1.04 (0.87-1.25)	.60	8.94	0.99 (0.83-1.17)	.90	8.92
More than 1occupant per room	1.12 (1.00-1.27)	.06	4.83	1.10 (0.98-1.23)	.10	4.71
No health insurance	1.02 (0.91-1.16)	.70	4.66	1.03 (0.91-1.16)	.70	4.68
Unemployment	1.01 (0.91-1.13)	.80	3.36	1.02 (0.91-1.14)	.70	3.36
COVID-19 case count	1.08 (0.97-1.21)	.10	2.94	1.09 (0.98-1.21)	.10	2.88

^a^IRR: incidence rate ratio.

^b^VIF: variance inflation factor.

## Discussion

### Principal Results

In this multi-scale analysis integrating and comparing spatial disease information from gold standard disease prevalence sources such as the US CDC, social determinants of health information from the US census, and satellite imagery data, we demonstrate an approach to identify characteristics of communities at risk for COVID-19 complications. We used the tools of unsupervised learning to develop a COVID-19 risk score that provides a single interpretable number that summarizes a communities’ (census tract) aggregate risk. The constituents of the COVID-19 risk score included census tract–level chronic disease risk factors that corresponded to previously identified individual-level risk factors for COVID-19, such as age, obesity, diabetes, and heart disease.

Others have deployed similar risk scores to identify communities at risk for COVID-19 [16] and have used social determinants of health to identify this risk [45-47]. Furthermore, we were inspired by the work of others that demonstrate how remote sensing images predict obesity prevalence [26]. However, to our knowledge, this is the first study to examine the relationship between COVID-19 risk in neighborhoods (quantified using the COVID-19 risk score) and the social determinants of health and satellite image information. We found that, by combining established social determinants, information measured on earth with the built environment from space can explain most of the variation in the COVID-19 risk score, with a mere 13 sociodemographic variables explaining 50% of variation and, when combined with satellite images, could explain ~90% of variation. As more COVID-19 data becomes available, this finding suggests that future risk models for COVID-19 could incorporate satellite imagery together with social determinants of health to better model risk. Currently, comprehensive measurement of the built environment is not typically used in the public health response to outbreaks, and COVID-19 pandemic risk models are typically modeled at the county level [46,47], a coarse geographical resolution that can obscure local hot spots or areas of need. Building models using the approach outlined here could help facilitate precision public health responses down to the local community (census tract) or subcensus tract level, thereby facilitating more precise allocations of resources to areas that need it.

Although it could be argued that the deep learning analysis of satellite imagery is simply a measurement of population density, this approach also measures several other factors that may contribute to COVID-19 infection and death rates independent of population density, such as built environment features that contribute to the development of COVID-19 risk factors and features that may put individuals at risk of contracting COVID-19. Examples of features that may put individuals at risk for developing risk factors include walkability (which contributes to obesity [48]) and road proximity (which can increase risk for heart disease [49]). Additionally certain architectural and built environment features that might put individuals at risk of COVID-19 infection, such as the configuration of pedestrian traffic in an urban area [50], can be partly quantified with this approach.

We believe that the COVID-19 risk score can be a tool in the growing armamentarium for public health and health care companies’ toolbox to enable communities to prepare for the potential onslaught of cases in the coming winter months, ultimately helping to “flatten the curve” [51] and achieve precision public health goals of improving local health. Notably, we found that the zip code–level COVID-19 risk score for New York City and surrounding areas predicted risk for COVID-19 complications such as death. Zip codes with the highest COVID-19 scores (in the top 5%) had double the risk of COVID-19 death versus zip codes with the lowest scores. As of this writing, New York City is contemplating another lockdown due to a surge in the same zip codes we identified as high risk [52]. Given the heterogeneity of various census tracts and neighborhoods across the United States and the range of COVID-19 rates and deaths, a more comprehensive national analysis will need to be performed using nationally representative comorbidity data and satellite data before extending the conclusions from the New York City analysis to similar jurisdictions in the United States or across the whole country.

As a byproduct of developing a risk score for communities, we observed that there is substantial variation of chronic disease prevalence within cities and across cities in the United States. With the exception of New York City and a few other places in the United States, public health agencies mostly collect COVID-19 case and death records at the county level across the country. However, the findings in our study implicate that smaller populations are at risk, and counties are heterogeneous.

We demonstrated how COVID-19 rates can be modeled using the COVID-19 risk score and how social determinants of health and the built environment can explain most of the score variance. Through simulations of the coprevalences of each of the 27,648 census tracts, we found that the point estimates for the community risk scores were robust to simulated sampling error. Many cities in the southwest and southeast demonstrated wide ranges in the COVID-19 risk score values. For example, Surprise, Arizona had a COVID-19 risk score with an IQR of 26 to 59. Atlanta, Georgia had an IQR of 24 to 41 (Figure S1 in Multimedia Appendix 2). Social determinants of health are hierarchical in structure and distributed over both geographic space and time whose measurement can occur on both the individual level (exposure of a person) or area level (exposure levels of a place). Satellite images provide a microscope into the area-level built environment, a concept that encapsulates the physical structures of how humans live, such as the city layout, resource presence, and landscape. A total of 65% of COVID-19 community risk score variance was explained by demographics and the social determinants of health, and 87% explained when the built environment was included. Given the large proportion of variance explained by the built environment, future precision public health strategies like hot spot identification and vaccine prioritization could be quickly improved by including measurements of the built environment to identify geographical areas in need of assistance.

This large proportion of COVID-19–associated risk variance explained by the social determinants of health and built environment may be partly due to how discrimination affects where people live, their built environment, and access to health care [15,53,54]. Since the built environment and social determinants of health were found to play an important role in explaining the variance associated with the COVID-19 risk score, we plan to integrate this information into future COVID-19 risk score calculations that can be extended across the United States beyond the 500 Cities data set. We found that ~90% of the variation of prevalence of the 15 disease and health indicator prevalences (eg, diabetes, obesity, cardiovascular disease, populations that take blood pressure medication, and average age) can be explained by just two dimensions.

### Limitations

The following are limitations of this study. First, we relied on disease and health indicator prevalence from the 500 largest cities in the United States but missed out on less urban areas whose populations are at risk for COVID-19 complications. In the future, we aim to task satellite imaging technology to locations that cannot be covered by resource-limited public surveillance programs. Second, although the CDC 500 Cities data are reflective of the diversity of individuals who live in a census tract, they are updated every 2 years and are dated to the latest collection (2019 data release reflects disease prevalence in 2017). Relatedly, neither individual-level disease nor COVID-19 status of individuals from these communities are measured. Third, satellite image data are captured at a resolution of approximately 20 m per pixel. It is not clear from our study if higher resolution images (that can theoretically capture more human-visible details of the built environment) would lead to better predictions of the COVID-19 risk score. Finally, interpretations of the New York–related data is limited due to the fact that it is aggregated to the zip code level. It is clear that COVID-19 is a disease of disparity; however, we cannot make a causal claim between the instruments such as the COVID-19 risk score, satellite imagery, and census tract–level sociodemographic factors, and eventual individual-level COVID-19–related complications.

### Conclusions

Although it is clear that individual-level comorbidities are associated with risk for COVID-19, here we show that communities’ clinical coprevalence structure are predictive of risk quantified by the COVID-19 risk score, and the variance of that score can be explained using the social determinants of health and the built environment measured from satellite imagery. We provide all our tools to monitor COVID-19 risk and related data in an interactive web-based dashboard.

## Appendix

Multimedia Appendix 1 Supplementary methods.

Multimedia Appendix 2 Supplementary information containing figures, tables, and application programming interface specification for the COVID-19 community risk score.

Multimedia Appendix 3 Table S2.

## Abbreviations

ACS: American Community Survey
CDC: Centers for Disease Control and Prevention
COPD: chronic obstructive pulmonary disease
IRR: incident rate ratio
MSE: mean squared error
VIF: variance inflation factor
ZCTA: zip code tabulation area
