# Supplementary material for: Identifying Communities at Risk for COVID-19–Related Burden Across 500 US Cities and Within New York City: Unsupervised Learning of the Coprevalence of Health Indicators
Source: JMIR Public Health Surveill. 2021 Aug 26;7(8):e26604. doi: 10.2196/26604 (PMC8396545; doi:10.2196/26604)
Supplement: Multimedia Appendix 1 [file publichealth_v7i8e26604_app1.docx]

## **Multimedia Appendix 1: Supplementary Methods**

### **Defining the COVID-19 Community Risk Score (COVID-19 Risk Score)**

To summarize the total variation of the disease prevalences in a single score, we scaled each of the 13 health indicators plus 2 indicators of males and females over 65 (z-score transformation) prevalence by subtracting the overall average and dividing by the standard deviation of the prevalence. Then, for each census tract, the z-scores were summed and rescaled to be between 0-100. Therefore, the tracts with the highest scores have the highest “additive” prevalences for all the health indicators.

Using the R programming language [1], the score utilized principal components analysis (PCA), a unsupervised machine learning approach that transforms the data (27,648 by 15 dimensions) into a new space where each new variable is a linear combination of variables from the original dataset. PCA attempts to maximize the variance explained over the dataset in successive new variables (denoted Y_1_, Y_2_Y_15_) that are defined by the “components”, or linear combination of each of the original variables (e.g., health indicators) (Figure 1E). Therefore, the first variables (Y_1_, Y_2_, etc) that correspond to the first principal components in the new dataset explain the maximal amount of variation in the entire dataset. After calculating the first two principal components using census tract data, denoted Y_1_ and Y_2_ (fitting each census tract to the first two principal components), we rescaled Y_1_ and Y_2_ (ie. multiplying the principal component by -1 when necessary) such that increasing values corresponded to increases in disease prevalences from the original 500 Cities data used as input. Next, we estimated a single score for each census tract as a weighted average between Y_1_ and Y_2_, where the weights were proportional to the variance explained by the first and second principal components respectively. Finally, the score was rescaled to a value between 0-100 using the “rescale” function from the R package scales (version 0.4.1). The higher the value, the higher the total burden of disease and proportion of individuals over 65 in that census tract. We calculated risk scores for different units of administrative areas, including the 500 cities, 316 counties, and 50 states. We estimated a city-wide prevalence of each of the 15 COVID-19 risk factors and diseases and then computed the additive and PCA-based scores as above. We repeated the same procedure for counties (m=316) and states (m=50) by recalculating the PCA at the county and state level.

Next, we sought to estimate how robust the PCA-based risk score is to sampling error via simulation. To do so, we estimated the standard deviation of the prevalences as a function of the size of the population of the tract. We assumed a covariance structure between the disease prevalences to be the observed census-level correlation across the US. Next, for each census tract, we simulated 100 times the prevalence of the diseases using a multivariate normal distribution, centered around the actual prevalence and with covariance equal to the COVID-19 risk factor correlation over all 27,648 tracts. Next, for each of the 100 simulated datasets we computed the principal components and obtained a simulated distribution about the principal component. Next, we estimated the predicted new projections for plus or minus 5 standard deviations (SD) of the principal components. The “robustness” score is the range of the score across the +/- 5 SD of the principal components.

### **Socioeconomic Correlates of the Community COVID-19 Risk Score**

For the linear regression analysis, we calculated the variance inflation factor (VIF) to examine multicollinearity using the “VIF” function in the regclass package (version 1.6) in R. We split the census tract dataset using a fully randomized approach into 50% training and 50% testing (for the random forest regression) to get a conservative estimate of variance explained and predictive capability of the sociodemographic variables in the COVID-19 Risk Score while not overfitting the data. To assess the robustness of these observations, the training/testing split was also performed using an 80% training and 20% testing fully randomized split. Specifically, we tested the linear and non-linear association prediction between American Community Survey 5-year proportions of individuals in each census tract who were (a) below poverty, (b) unemployed, (c) non-employed, (d) have less than high school education, (e) lack health insurance, (f) have more than one person occupied per room, and (g) Hispanic, Asian, African American, or Other Ethnic group. Furthermore, we also included the median income and median home value of a census tract. Coefficients in the linear regression denoted a 1 unit change in a 1 SD change in the socioeconomic variable (e.g., a 1 SD unit change in the prevalence or a 1 SD change in the median income for a census tract). Random forests were fit using 1000 trees and a tree size of 5.

### **The Community COVID-19 Risk Score Application Programming Interface and Dashboard**

To distribute the data to other software applications, users, and provide interactive graphing capabilities, we created an Application Programming Interface (API) and dashboard for the COVID-19 Risk Score. We obtained COVID-19 case data from Johns Hopkins University [2]. The API endpoints and their outputs are described in Appendix I. We also developed a dashboard through the API in the form of geographical coordinate information for Census tracts, and data values (in GeoJSON format) to construct a choropleth in the web browser. We obtained geographical coordinates and shapefiles from the US Census (2018) [3] . Distributions of values are displayed for Census tracts being viewed at a particular location (the interactive dashboard is currently available online [4] (see a screenshot in the Supplementary Information). Our code and data are available as open-source under the MIT license [5].

## References

1 R Foundation for Statistical Computing, Vienna, Austria. A language and environment for statistical computing [Internet]. 2017. Available from: http://www.R-project.org/

2 COVID-19 [Internet]. Github; [cited 2020 Oct 4]. Available from: https://github.com/CSSEGISandData/COVID-19

3 United States Census Bureau. Cartographic boundary files - shapefile [Internet]. [cited 2020 Dec 17]. Available from: https://www.census.gov/geographies/mapping-files/time-series/geo/carto-boundary-file.html

4 XYHealth. XY.ai COVID-19 Dashboard [Internet]. XY.ai COVID-19 Dashboard. [cited 2021 Feb 1]. Available from: https://covid19satellite.org/

5 Patel CJ. covid_comorbidity_score [Internet]. Github; [cited 2020 Dec 16]. Available from: https://github.com/xyhealth/covid_comorbidity_score
